# Relationship between Motor and Nonmotor Symptoms and Quality of Life in Patients with Parkinson’s Disease

**DOI:** 10.3390/nursrep12010001

**Published:** 2021-12-24

**Authors:** Eduardo Candel-Parra, María Pilar Córcoles-Jiménez, Victoria Delicado-Useros, Antonio Hernández-Martínez, Milagros Molina-Alarcón

**Affiliations:** 1 Department of Nursing, Physiotherapy and Occupational Therapy, Faculty of Nursing, University of Castilla-La Mancha, Av. de España, s/n, 02001 Albacete, Spain; Eduardo.Candel@uclm.es (E.C.-P.); pilar.corcoles@uclm.es (M.P.C.-J.); victoria.delicado@uclm.es (V.D.-U.); 2 Instituto de Investigación en Discapacidades Neurológicas (IDINE), University of Castilla-La Mancha, Av. de España, s/n, 02001 Albacete, Spain

**Keywords:** Parkinson’s disease, quality of life, PDQ-39, motor symptoms, nonmotor symptoms

## Abstract

Background: Parkinson’s disease (PD) is a chronic neurodegenerative disease that implies a progressive and invalidating functional organic disorder, which continues to evolve till the end of life and causes different mental and physical alterations that influence the quality of life of those affected. Objective: To determine the relationship between motor and nonmotor symptoms and the quality of life of persons with PD. Methods: An analytic, descriptive, cross-sectional study was conducted with patients with different degrees of PD in the Albacete Health district. The estimated sample size required was 155 patients. The instruments used for data collection included a purpose-designed questionnaire and “Parkinson’s Disease Questionnaire” (PDQ-39), which measures eight dimensions and has a global index where a higher score indicates a worse quality of life. A descriptive and bivariate analysis was conducted (SPSS^®^ IBM 24.0). Ethical aspects: informed consent and anonymized data. Results: A strong correlation was found between the number of motor and nonmotor symptoms and global health-related quality of life and the domains mobility, activities of daily living, emotional well-being, cognitive status, and pain (*p* < 0.05). Receiving pharmacological treatment and taking more than four medicines per day was significantly associated with a worse quality of life (*p* < 0.05). Patients who had undergone surgical treatment did not show better global quality of life (*p* = 0.076). Conclusions: All nonmotor symptoms and polypharmacy were significantly associated with a worse global quality of life.

## 1. Introduction

Parkinson’s disease (PD) is the second most frequent neurodegenerative disease after Alzheimer’s disease. Its diagnosis has a considerable impact on the health-related quality of life (HRQoL). It causes around 4600 deaths annually (11th on the list of causes of death, 1.1% of the total), and its mortality rate has increased over the last two decades [1]. In Spain, the most recent statistics indicate that there are between 7000 and 10,000 affected. The estimated incidence in Spain is between 20 and 25 new cases per year for every 100,000 people. Hospital clinical records indicate a worldwide prevalence of 100–300 cases per 100,000 people. PD occurs more in men than in women, and the cause of this uneven incidence remains unknown [2,3]. Pathologically, PD is defined by the progressive loss of dopaminergic neurons in the substantia nigra and the presence of inclusions called Lewy bodies. This neuronal death leads to a dopamine deficit in the striatal endings that transmit information for the correct control of movements. The underlying cause of PD, as for most neurodegenerative diseases, remains unknown, although it is probably multifactorial, including factors related to the individual’s own biological, genetic, environmental, and aging characteristics [4,5,6]. No biological markers are known for its diagnosis. Hence, the diagnosis of PD is clinical and is established from the medical history and examination [5].

From a clinical point of view, two large groups of disorders are distinguished. The first refers to motor symptoms (MS)—tremor, poor or slow movements (akinesia), increased muscle tone or rigidity, and abnormal involuntary movements (dyskinesias). The association of tremor, rigidity, akinesia–bradykinesia, and alteration of postural reflexes constitutes the so-called “Parkinsonian syndrome”. More than 40% of those affected have postural and action tremor, although between 10–25% do not experience any tremor. Slow movement and stiffness limit normal function, with stiffness resulting in muscle pain in these patients. However, the most serious motor problem is akinesia, with varying degrees of movement and changes in position [3,5].

The other group of disorders refers to nonmotor symptoms (NMS), with manifestations that indicate the existence of autonomic dysfunction (constipation, hyperhidrosis), sensory dysfunction (paresthesia, pain), and psychiatric problems (depression, dementia) [4].

Mood and cognitive disorders affect 80.6% of these patients [7,8] and have a severe negative effect on perceived HRQoL [9]. Depression (associated or not with anxiety) is the neuropsychiatric disorder that has the greatest influence on decreasing HRQoL in these patients [8,10]. These symptoms are directly associated with HRQoL regardless of the severity of MS. The variables that are considered prognostic include depressive symptoms, insomnia, and a low degree of independence in relation to the severity of the disease. Half of patients report a lack of energy, related to depressive symptoms [11,12].

To classify the evolution and progression of this disease, the Hoehn and Yahr (HY) stages are used, which are based on motor alterations and identify five stages from the least to the most affected [13].

To date, there is no curative treatment for PD, so treatment focuses on improving symptoms, delaying motor complications, and prolonging autonomy for as long as possible. Pharmacological, surgical, and rehabilitative treatments can be used, and they can be combined. Levodopa and other dopamine agonist drugs are used as pharmacological treatments. Surgical techniques such as deep brain stimulation (DBS) can, in some cases, improve PD symptoms. Some studies indicate a slight improvement in HRQoL in operated patients [14,15].

Chronic diseases affect all aspects of an individual’s life and generally combine a wide variety of elements that influence well-being and satisfaction with life. HRQoL is understood as the perception and evaluation by the patients themselves of the impact caused in their lives by the disease and its consequences; it includes physical, mental, and social aspects related to the state of health and care, in addition to the global perceptions about health and other personal constructs.

In the absence of a cure, one of the main goals of care is to improve or maintain the patient’s HRQoL [16].

The present study aimed to analyze how the different motor and nonmotor symptoms in persons with Parkinson’s disease affect their quality of life.

## 2. Materials and Methods

**Design:** Analytic observational cross-sectional study.

**Study population:** Patients diagnosed with PD and seen at the Movement Disorders Unit of the Neurology Service of the Albacete Integrated Management Area.

**Inclusion criteria:** All the patients diagnosed with PD seen by the Movement Disorders Unit who voluntarily agreed to participate in this study. The patients that did not understand Spanish were excluded. All had to have a contact address, telephone and no cognitive problems that prevented them from understanding the questionnaire.

**Sample size:** For an estimated prevalence of PD in the general population of 187 cases per 100,000 inhabitants [7] and with a population of 414,892 in the Albacete Health District in 2015, around 776 persons diagnosed with PD were expected. If the mean score and standard deviation of HRQoL were similar to that obtained in the Rahman study (mean, 32.4; SD = 16.3) [17], for a confidence level of 95% and a precision of ± 2.5, the estimated sample size would be 135 participants (EpiDat v3.1.; Epidemiology Service of the Directorate-General of Public Health, Santiago de Compostela, Spain). The above sample size was increased by 15% for possible non-responders, giving a total sample of 155 participants. Participants were recruited consecutively until the estimated sample size was reached. The participants were recruited from January 2015 to December 2016.

**Measuring instruments:** “Parkinson’s Disease Questionnaire” (PDQ-39) [18] was used to measure HRQoL. PDQ-39 contains 39 items, which cover eight dimensions. These dimensions are mobility, activities of daily living (ADL), emotional well-being, social support, stigma, communication, cognitive state, and pain. Each item has five options (from 0 = never to 4 = always or unable to do). The results were calculated as percentages, adding the scores for the items in each dimension, multiplying by 100, and dividing by the maximum score of each dimension. A higher score indicates a poorer HRQoL. A global index can be obtained by taking the mean of the scores for each dimension (PDQ Summary Index, PDQ-39 SI) [18] that summarizes the result of the scale. This questionnaire has been used in clinical trials, in which the variations in the different dimensions have been congruent with the clinical evaluations made using the usual scales for PD, supporting its adequate sensitivity to change in the clinical features of the patients.This questionnaire was completed through a personal interview at the time of recruitment.

**Study variables:** Study variables included sociodemographic variables (age, sex, marital status, living situation, employment status, education level) and clinical variables (HY stages, PD duration, measured from the date of diagnosis to the date of inclusion in the study; other previously diagnosed pathologies; performance of deep brain stimulation (DBS); presence of MS (five items) and NMS (10 items); apathy, depression; drug treatment and nondrug treatment).

In addition, social and health variables were collected, such as existence of a caregiver, degree of disability (measured according to current regulations in Spain) [19], care provisions for dependency, and mortality. The primary outcome variable was HRQoL.

**Study procedures**: First, before the data collection phase, we conducted a pilot study with ten patients to assess the validity of the purpose-made data collection form. An information sheet was prepared outlining the study’s objectives, and written consent was requested for patients who wished to participate in the study. Data collection was carried out in the Movement Disorder Unit once written informed consent was obtained. Next, an interview was carried out with the patients and caregivers, and the clinical history since diagnosis was consulted. The interviewers were nurses who had received interviewing training. Then, the evaluators collected the PDQ-39 questionnaire responses, and later the investigators processed the data.

**Statistical analysis:** Statistical software IBM SPSS v.24 (IBM, Armonk, NY, USA) was used for statistical analyses. A descriptive statistical analysis was performed for each variable, calculating the absolute and relative frequencies for the qualitative variables and the mean and standard deviation (SD) for quantitative variables. The Kolmogorov–Smirnov and Levene’s tests were used to check the fit of the empirical data with normal distribution and the homoscedasticity of the distributions; 95% confidence intervals (CI) were calculated, and *p* < 0.05 was considered statistically significant. Next, a bivariate analysis was performed, in which the outcome variable was HRQoL (both the global score using the PDQ-39 SI and each different dimension of PDQ-39) and the different factors theoretically related to it (clinical and sociodemographic variables). Depending on the type of variables analyzed, Pearson’s chi-squared test (χ^2^), and Fisher’s exact test when appropriate, Student’s *t*-test, and ANOVA (post hoc tests included Scheffe’s test when homoscedasticity could be assumed, and the Games–Howell test otherwise), as well as the Pearson correlation coefficients (r) (coefficient values < 0.3 were considered as a weak correlation, between 0.3 and 0.50—a moderate correlation, and ≥ 0.5—a strong correlation) were used [20].

## 3. Results

Finally, 155 participants with valid data were included. The main characteristics of the sample are shown in Table 1.

Medication data were collected via a purpose-designed survey filled out by patients and caregivers and later verified in the clinical history. Therefore, it was the UTM neurologists that calculated the doses and the medication taken by the patients.

In terms of PD symptoms, the most frequent MS were slowness of movement (88.4% (137)), postural instability (61.9% (96)), tremor (52.3% (81)), difficulty turning (45.8% (71)), and dyskinesias (29% (45)). Among the NMS, fatigue was present in 67.1% of the paticipants (104), pain—in 58.1% (90), daytime sleepiness—in 54.8% (85), urinary incontinence—in 51% (79), depression/apathy—in 49% (76), constipation—in 48.4% (75), insomnia—in 41.3% (64), dysphagia—in 35.5.% (55), delayed gastric emptying—in 27.7% (43), and esophageal dysmotility—in 14.8% (23). The mean MS present in patients was 2.77 (SD = 1.26; 95% CI: 2.57–2.97), and for the NMS it was 4.47 (SD = 2.24; 95% CI: 4.12–4.83); for the MS and NMS present at the same time it was 7.25 (SD = 3.09; 95% CI: 6.76–7.74).

Below, we present the HRQoL of the patients according to the HY stages (1, 2, 3, 4, 5) as these stages are defined based on different mobility factors. In the initial HY stages (1, 2) and the more advanced stages (3, 4, 5) in the different dimensions of the PDQ-39 questionnaire, a statistically significant relationship was found with a worse HRQoL both in the global PDQ-39 SI score and in the majority of its dimensions (Table 2). Of note, mobility and ADLs considerably decreased HRQoL between the initial and advanced stages, while the differences found in the social support dimension were minimal and not statistically significant.

Next, we examined the relationship between the number of MS, NMS, and both combined (MS–NMS) and global HRQoL and each dimension of PDQ-39 using Pearson’s correlation (r). A strong correlation was found between the number of MS and global HRQoL and the dimensions of mobility, ADLs, cognitive status, and pain, whereas the correlation was moderate for the emotional well-being and communication dimensions and weak for the dimensions of stigma and social support. A greater number of MS and NMS, both separately and together (MS + NMS), was also correlated with a higher score in the PDQ-39 SI, which implies a worse HRQoL (Table 3).

Regarding the presence of MS, such as slowness of movement, postural instability, difficulty turning, and dyskinesias, all were statistically significantly related with a worse HRQoL, except for tremor, which showed no difference in HRQoL between those that had or did not have tremor. This result should be highlighted, as tremor is considered very characteristic in these patients (Table 4).

The presence of NMS was also statistically significantly related to a worse global HRQoL (PDQ-39 SI) (Table 5). The biggest differences were found for fatigue, apathy/depression, pain, esophageal dysmotility, urinary incontinence, and insomnia. We highlight pain, urinary incontinence, and digestive difficulties (constipation, dysphagia, delayed gastric emptying, and altered esophageal motility) owing to the importance patients place on these aspects and the possibility of interventions to improve them.

Below, the relationship of MS and NMS with the recoded HY stages is shown (Table 6).

Considering that DBS is a treatment that attempts to improve some of the main PD symptoms, we believed it would be interesting to evaluate the different HRQoL in these patients. Unfortunately, no statistically significant relationship was found in global HRQoL between the patients who had undergone DBS and those who had not. Nonetheless, a statistically significant relationship did exist in the dimensions of mobility, ADLs, and communication (Table 7).

The MS and NMS related to PD are often treated with medicines, with NMS resulting in greater consumption of medicines in the early stages. Patients with polypharmacy (more than four medicines) have a statistically significant worse HRQoL (Table 8).

## 4. Discussion

In the present study, we aimed to analyze how different motor and nonmotor symptoms in patients with PD influenced their quality of life. The global HRQoL of the patients included in our study indicated that it was partially affected, even without severe PD problems, with a mean PDQ-39 score lower than 30 [20,21,22].

The number of MS had a statistically significant relationship with a worse HRQoL. According to the symptoms that a patient had, they had a different score for global HRQoL. We observed that a statistically significant relationship existed with HRQoL for difficulty turning, dyskinesias, postural instability, and slowness of movement. In our study, the mean number of MS that a patient had was 2.77, with the most frequent being slowness of movement, postural instability, and difficulty turning—those that cause a worse HRQoL in these patients. Nevertheless, tremor, which was significantly associated with HRQoL in studies by Hassan et al., 2012, and Martínez Fernández et al., 2016, was not significantly associated with a worse HRQoL in our results [20,23]. In our study, tremor came fourth in the list of MS that patients considered to affect their HRQoL. This discrepancy with other studies is likely due to the influence of PD onset at an early age as this group of patients was less than 20% in our study.

Regarding NMS, the mean number of symptoms in our patient sample was 4.47. In addition, we observed a statistically significant relationship between the HRQoL scores and all the NMS. These results agree with those obtained by Erro et al., 2016, and Prakash et al., 2016, where the total number of NMS significantly predicted the HRQoL score and had a greater influence than the motor scores [21,24]. Therefore, NMS play a key role in the clinical aspect of this disease and must be addressed specifically in clinical trials with specific scales. Along these lines, D’Iorio et al., 2017 indicated that anxiety, depression, apathy, associated with a decrease in attention, significantly reduce autonomy and HRQoL. Therefore, early identification and management of these neuropsychiatric symptoms should be relevant to preserve HRQoL in PD [25]. Concerning other NMS, such as esophageal dysmotility, dysphagia, choking, respiratory difficulties, urinary incontinence, constipation, and delirium, our results also coincide with the study by Conran et al., 2019 [26]. Likewise, for fatigue, Sheard et al., 2014, concluded that fatigue is a common symptom in these patients and is closely related to HRQoL [27].

A higher number of MS and NMS, separately or combined (MS + NMS), implies a higher score on the PDQ-39 SI and, therefore, a worse overall HRQoL. In our study, the mean number of MS + NMS was 7.25 symptoms per patient. Navarta-Sánchez et al., 2016, reported that the main factors associated with poor HRQoL were motor impairment and the number of NMS [28].

The current clinical practice does not address the need to include scores for the domains that assess NMS when evaluating PD, despite their considerable influence on disability and HRQoL. However, taking into account our results, the evaluation of NMS should be carried out in combination with MS. Duncan et al., 2014, noted that the detection and recognition of NMS have been gaining relevance in recent years due to their high prevalence with the progress of the disease, producing a negative impact on the HRQoL of patients to a greater degree than MS [29]. Martínez-Martin, 2017, wrote that PD involves changes in patients both due to MS (difficulties in movement, slowness, etc.) and NMS, such as mood disorders (depression and anxiety, concentration difficulties), disability (difficulties with personal grooming, falls, communication problems), and social dysfunction (limitations in social activities, stigma); all causing a high level of stress in patients [8]. Prakash et al., 2016, concluded that, unlike motor disabilities, the burden of NMS, particularly sleep and mood, has a significant impact on the global HRQoL of PD patients in a two-year follow-up period [24].

In our study, the patients were treated by using drugs to alleviate the effect of MS and NMS. Thus, patients with polypharmacy (more than four drugs) have a statistically significantly worse HRQoL. In addition, in the study by Martínez-Fernández et al., 2016, the authors observed that pharmacological therapy has adverse effects such as headaches, nausea, dry mouth, constipation, drowsiness, fatigue, dizziness, nightmares, hypotension, and motor fluctuations [30]. There is consensus that pharmacological treatment with levodopa improves severe motor disorders such as dyskinesias. However, when these treatments do not work, there is a need to consider DBS. Schuepbach et al., 2013, stated that subthalamic stimulation reduces motor disability and improves HRQoL in patients with advanced PD who have a severe motor disorder induced by complications of levodopa. In addition, they noted that subthalamic stimulation is more effective than medical therapy in patients with PD with early motor complications [31]. However, there is a possibility that some adverse reactions to the medication will manifest themselves in the progress of the illness in patients. These issues remain unsolved and need to be investigated further in future studies.

In our society, with many older adults and a significant number of patients with PD, an effort should be made to develop health policies that improve the HRQoL, which would benefit families and patients and reduce health costs.

### 4.1. Implications for Clinical Practice

The definition of clinical stages is based mainly on motor symptoms; however, the influence of NMS on global HRQoL indicates they should be included in the assessment of patients with PD. Many NMS are preventable or treatable with nursing interventions within the framework of the multidisciplinary team; therefore, this provides a pathway for nursing action to improve the HRQoL of patients with PD through therapeutic interventions for NMS. Interventions to prevent or treat constipation, urinary incontinence, and dysphagia, as well as educational interventions and training programs aimed at both patients and caregivers, can be carried out autonomously by nurses in the community setting or other settings.

Regarding the finding of a worse HRQoL for the mobility, pain, and affective-emotional dimensions in women, we consider that interventions specifically aimed towards women should be developed to manage the gender-associated issues, which would lead to an improvement in the global HRQoL.

Another priority area of intervention revealed in this study is pharmacological management which, from the nursing point of view, could imply a review of the prescribed medication to detect possible duplications and adverse effects. Likewise, consultation with the responsible doctor should be considered to assess whether it is possible to withdraw any drug, thus preventing or avoiding polypharmacy.

Given that pain is one of the dimensions of HRQoL that shows the greatest deterioration, interventions aimed at its relief and prevention should be considered a priority and relevant in patients with PD, especially since adequate pain management would impact the improvement of HRQoL. From the nursing point of view, interventions aimed at assessing pain should be reinforced using appropriate scales, adding nonpharmacological interventions for its control (relaxation, environmental management, among others), and collaborating with the multidisciplinary team to ensure adequate pharmacological treatment.

### 4.2. Limitations

Individuals in the study sample were patients under the care of the UTM of the Neurology Service of the Integrated Management Area of Albacete, a referral center for patients from other provinces of the autonomous community of Castilla-La Mancha. It is possible that the patients referred from other provinces had worse HRQoL because they were in more advanced stages of their illness and, because of the complexity of their situation, they could not be treated in their hospital of origin. Furthermore, patients with deep brain stimulation were slightly overrepresented in our sample, with 20.6% of the studied population having a surgically implanted neurostimulator. Finally, due to the consecutive selection of the sample until the sample size of 155 patients was met, it is possible that the sample was affected by recruitment bias.

It is possible that recruitment through face-to-face appointments at the Movement Disorder Unit of the Neurology Service of the Integrated Management Area of Albacete reduced the inclusion in the study of patients in the most advanced stages as having worse mobility conditions may have made it difficult for them to travel to the hospital for an in-person consultation. For this reason, the number of patients captured in the most advanced stage of HY was very small, which would limit the generalizability of the results in this subgroup of patients. The sociodemographic and social health variables were self-reported by the subjects, therefore, there could be some memory bias or concealment of sensitive data.

## 5. Conclusions

The presence of MS and NMS is associated with a worse overall HRQoL. All MS worsen HRQoL, specifically, difficulty turning, dyskinesias, and postural instability. Dyskinesias affect not only mobility but also emotional well-being, communication, and pain. NMS such as fatigue, pain, dysphagia, urinary incontinence, insomnia, and depression are perceived by patients as those symptoms that produce the worst HRQoL. Furthermore, the presence of more than five NMS is correlated with a worse overall HRQoL.

## Figures and Tables

**Table 1:** Sociodemographic and clinical characteristics of the study sample.

Variable	% (*n*) *N* = 155
Mean age (SD) 95% CI	69.51 (8.63) 68.14–70.88
Sex	
Male	59.4 (92)
Female	40.6 (63)
Living situation	
In a family	85.8 (133)
Lives alone	9.7 (15)
In a residence	4.5 (7)
Civil status	
Married	75.5 (117)
Widowed	16.1 (25)
Divorced	1.9 (3)
Employment situation	
Retired	73.5 (114)
Housework	19.4 (30)
Actively employed	5.2 (8)
Education	
No education	0.6 (1)
Primary level	82.6 (128)
Secondary level	12.3 (19)
University level	4.5 (7)
HY stage	
Stage 1, 2	67.1 (104)
Stage 3, 4, 5	32.9 (51)
Surgery for neurostimulation	
Yes	20.6 (32)
No	79.4 (123)
Medicines consumption	
≥4 medicines	41.3 (64)
<4 medicines	58.7 (91)

SD, standard deviation; CI, confidence interval.

**Table 2:** Differences between the total scores of the PDQ-39 domains based on the recoded HY stages.

	Score Mean (SD) 95% CI	HY Stage	Mean (SD)	Mean Difference	95% CI	*p*
Total PDQ-39 SI	27.47 (16.14) 24.91–30.03	Stage 1, 2	20.17 (11.97)	−22.2	−26.3, −18.03	0.001 *
		Stage 3, 4, 5	42.37 (13.06)			
Total mobility	36.08 (30.18) 29.99–39.97	Stage 1, 2	21.92 (20.65)	−43.02	−50.6, −35.45	0.001 *
		Stage 3, 4, 5	64.95 (25.69)			
Total ADL	32.04 (27.71) 25.82–34.76	Stage 1, 2	19.79 (19.29)	−37.23	−44.5 −29.96	0.001 *
		Stage 3, 4, 5	57.02 (25.48)			
Total emotional well-being	36.23 (24.03) 32.23–40.05	Stage 1, 2	30.32 (23.60)	−17.95	−25.5, −10.33	0.001 *
		Stage 3, 4, 5	48.28 (20.27)			
Total stigma	19.23 (24.55) 15.33–23.13	Stage 1, 2	13.64 (20.13)	−16.99	−24.8, −9.13	0.001 *
		Stage 3, 4, 5	30.63 (28.70)			
Total social support	18.49 (18.54) 15.55–21.43	Stage 1, 2	16.74 (16.92)	−5.31	−11.5, −0.91	0.094
		Stage 3, 4, 5	22.05 (21.2)			
Total cognitive status	29.27 (20.95) 25.94–32.59	Stage 1, 2	22.71 (18.47)	−19.93	−26.2, −13.58	0.001 *
		Stage 3, 4, 5	42.64 (19.43)			
Total communication	16.07 (19.99) 12.9–19.24	Stage 1, 2	9.21 (13.97)	−20.85	−26.7, −14.95	0.001 *
		Stage 3, 4, 5	30.06 (23.03)			
Total pain	32.36 (23.02) 28.71–36.01	Stage 1, 2	27.00 (21.42)	−16.29	−23.6, −8.78	0.001 *
		Stage 3, 4, 5	43.3 (22.48)			

Purpose-made questionnaire. The results are shown as the means and standard deviation. * Statistical significance. Abbreviations: ADL, activities of daily living; HY, Hoehn and Yahr.

**Table 3:** Analysis of the relationship between the number of symptoms with the PDQ-39 SI and total PDQ-39 in each dimension.

*N* = 155	No. of MS		No. of NMS		No. of MS + NMS	
	Pearson’s Correlation (*r*)	*p*	Pearson’s Correlation (*r*)	*p*	Pearson’s Correlation (*r*)	*p*
PDQ-39 SI	0.705	0.001 *	0.732	0.001 *	0.732	0.001 *
Mobility	0.534	0.001 *	0.528	0.001 *	0.603	0.001 *
ADLs	0.531	0.001 *	0.473	0.001 *	0.562	0.001 *
Emotional well-being	0.352	0.001 *	0.567	0.001 *	0.557	0.001 *
Stigma	0.177	0.028 *	0.319	0.001 *	0.305	0.001 *
Social support	0.131	0.105	0.187	0.020 *	0.189	0.018 *
Cognitive state	0.404	0.001 *	0.565	0.001 *	0.577	0.001 *
Communication	0.343	0.001 *	0.354	0.001 *	0.398	0.001 *
Pain	0.401	0.001 *	0.543	0.001 *	0.559	0.001 *

* Statistical significance.

**Table 4:** Relationship between the PDQ-39 SI and the presence of MS.

Motor Symptoms		*n* (%)	PDQ-39 SI Score Mean (SD)	Mean Difference	95% CI	*p*
Slowness of movement	Yes	137 (88.4)	29.44 (15.65)	16.92	9.37, 24.48	0.001 *
	No	18 (11.6)	12.51 (11.55)			
Postural instability	Yes	96 (61.9)	32.8 (15.47)	14.00	9.2, 18.8	0.001 *
	No	59 (38.1)	18.8 (13.3)			
Difficulty turning	Yes	71 (45.8)	35.8 (13.35)	15.37	10.83, 19.91	0.001 *
	No	84 (54.2)	20.43 (14.95)			
Tremor	Yes	81 (52.3)	27.96 (16.32)	1.01	−4.12, 6.16	0.697
	No	74 (47.7)	26.94 (16.04)			
Dyskinesias	Yes	45 (29)	34.5 (15.5)	9.71	4.26, 15.15	0.001 *
	No	110 (71)	24.65 (15.61)			

* Statistical significance.

**Table 5:** Association between the PDQ-39 SI and the presence of NMS.

Nonmotor Symptoms		*n* (%)	Mean (SD)	Mean Difference	95% CI	*p*
Esophageal dysmotility	Yes	21 (14.2)	37.5 (15.23)	11.78	4.79, 18.76	0.001 *
	No	127 (85.8)	25.72 (15.71)			
Apathy/depression	Yes	78 (49.0)	34.8 (15.22)	14.37	9.77, 18.97	0.001 *
	No	79 (51.0)	20.42 (13.76)			
Insomnia	Yes	64 (41.3)	33.47 (16.77)	10.22	5.26, 15.18	0.001 *
	No	91 (58.7)	23.25 (14.33)			
Dysphagia	Yes	55 (35.5)	33.1 (15.00)	8.73	3.54, 13.91	0.001 *
	No	100 (64.5)	24.37 (15.98)			
Pain	Yes	90 (58.1)	33.2 (15.08)	13.66	8.93, 18.39	0.001 *
	No	65 (41.9)	19.54 (14.17)			
Urinary incontinence	Yes	79 (51.0)	32.7 (15.46)	10.68	5.83, 15.53	0.001 *
	No	76 (49.0)	22.02 (15.09)			
Fatigue	Yes	104 (67.1)	32.46 (15.01)	15.16	10.25, 20.06	0.001 *
	No	51 (32.9)	17.30 (13.47)			
Delayed gastric emptying	Yes	43 (27.7)	31.79 (16.37)	5.98	0.32, 11.64	0.038 *
	No	112 (72.3)	25.81 (15.82)			
Constipation	Yes	75 (48.4)	31.12 (14.72)	7.06	2.04, 12.08	0.006 *
	No	80 (51.6)	24.05 (16.75)			
Daytime sleepiness	Yes	85 (54.8)	31.09 (15.87)	8.01	2.99, 13.00	0.002 *
	No	70 (45.2)	23.08 (15.48)			

* Statistical significance.

**Table 6:** Relationship between MS and NMS and the recoded HY stages.

		Stage 3, 4, 5 % (*n*)	Stage 1, 2 % (*n*)	(χ^2^)	*p*	OR	95% CI	
Slowness of movement	Yes	35.04 (48)	64.96 (89)	2.431	0.118	2.69	0.74	9.78
	No	5.88 (3)	14.42 (15)					
Tremor	Yes	34.57 (28)	65.43 (53)	0.213	0.644	1.17	0.59	2.29
	No	31.08 (23)	68.92 (51)					
Postural instability	Yes	45.83 (44)	54.17 (52)	19.099	0.001 *	6.28	2.59	15.23
	No	11.86 (7)	88.14 (52)					
Difficulty turning	Yes	59.15 (42)	40.85 (29)	40.896	0.001 *	12.06	5.22	27.89
	No	10.71 (9)	89.29 (75)					
Dyskinesias	Yes	57.78 (26)	42.22 (19)	17.771	0.001 *	4.65	2.21	9.75
	No	22.73 (25)	11.27 (85)					
Relationship between NMS and the recoded HY stages								
Fatigue	Yes	42 (40.38)	62 (59.62)	8.013	0.004 *	3.16	1.39	7.17
	No	9 (17.65)	42 (82.35)					
Pain	Yes	39 (43.33)	51 (56.67)	10.575	0.001 *	3.37	1.59	7.16
	No	12 (18.46)	53 (81.54)					
Daytime sleepiness	Yes	36 (42.35)	49 (57.65)	7.61	0.005 *	2.69	1.31	5.5
	No	15 (21.43)	55 (78.57)					
Apathy/depression	Yes	31 (40.79)	45 (59.21)	4.2	0.04 *	2.03	1.02	4.02
	No	20 (25.32)	59 (74.68)					
Urinary incontinence	Yes	35 (44.3)	44 (55.7)	9.48	0.002 *	2.98	1.46	6.05
	No	16 (21.05)	60 (78.95)					
Constipation	Yes	34 (45.33)	41 (54.67)	10.16	0.001 *	3.07	1.52	8.2
	No	17 (21.25)	63 (78.75)					
Dysphagia	Yes	25 (45.45)	30 (54.55)	6.08	0.013 *	2.37	1.18	4.74
	No	26 (26)	74 (74)					
Insomnia	Yes	31 (48.44)	33 (51.56)	11.91	0.001 *	3.33	1.66	6.69
	No	20 (21.98)	71 (78,02)					
Delayed gastric emptying	Yes	18 (41.86)	25 (58.14)	2.16	0.141	1.73	0.83	3.57
	No	33 (29.46)	79 (70.54)					
Esophageal dysmotility	Yes	12 (52.17)	11 (47.83)	4.54	0.033 *	2.6	1.05	6.39
	No	39 (29.55)	93 (70.45)					

Purpose-made. * Statistical significance.

**Table 7:** Differences among the total scores for the PDQ-39 domain scores in the patients who had and had not undergone DBS.

	DBS Treatment	Mean (SD)	Mean Difference	95% CI	*p*
Total PDQ-39 SI	Yes	31.98 (14.78)	5.67	−0.60, 11.96	0.076
	No	26.3 (16.33)			
Total mobility	Yes	49.21 (30.42)	16.55	4.98, 28.12	0.005 *
	No	32.66 (29.28)			
Total AVD	Yes	42.44 (27.68)	13.11	2.41, 23.8	0.017 *
	No	29.33 (27.18)			
Total emotional well-being	Yes	39.58 (24.11)	4.21	−5.21, 13.64	0.378
	No	35.36 (24.03)			
Total stigma	Yes	20.31 (24.78)	1.35	−8.29, 11.01	0.781
	No	18.95 (25.07)			
Total social support	Yes	19.53 (18.64)	1.3	−5.98, 8.59	0.724
	No	18.22 (18.58)			
Total cognitive status	Yes	29.29 (18.97)	0.28	−8.21, 8.27	0.995
	No	29.26 (21.53)			
Total communication	Yes	26.56 (22.24)	13.21	5.64, 20.78	0.001 *
	No	13.34 (18.5)			
Total pain	Yes	28.9 (21.48)	−4.35	−13.38, 4.67	0.342
	No	33.26 (23.41)			

* Statistical significance.

**Table 8:** Influence of polypharmacy on HRQoL.

Factors		PDQ-39 SI Mean (SD)	Mean Difference	95% CI	*p*
Number of medicines	≥4	33.68 (16.9)	10.58	15.52, 5.64	0.001 *
	<4	23.10 (14.12)			

* Statistical significance.

## Data Availability

The datasets generated and/or analyzed during the current study are available from the corresponding author on reasonable request. The preparation of this manuscript meets the requirements of the STROBE statement.
